# Surround Modulation Characteristics of Local Field Potential and Spiking Activity in Primary Visual Cortex of Cat

**DOI:** 10.1371/journal.pone.0064492

**Published:** 2013-05-15

**Authors:** Li Zhang, Bing Li

**Affiliations:** 1 State Key Laboratory of Brain and Cognitive Sciences, Institute of Biophysics, Chinese Academy of Sciences, Beijing, China; 2 University of Chinese Academy of Sciences, Beijing, China; University of Salamanca- Institute for Neuroscience of Castille and Leon and Medical School, Spain

## Abstract

In primary visual cortex, spiking activity that evoked by stimulus confined in receptive field can be modulated by surround stimulus. This center-surround interaction is hypothesized to be the basis of visual feature integration and segregation. Spiking output has been extensively reported to be surround suppressive. However, less is known about the modulation properties of the local field potential (LFP), which generally reflects synaptic inputs. We simultaneously recorded spiking activity and LFP in the area 17 of anesthetized cats to examine and compare their modulation characteristics. When the stimulus went beyond the classical receptive field, LFP exhibited decreased power along the gamma band (30–100 Hz) in most of our recording sites. Further investigation revealed that suppression of the LFP gamma mean power (gLFP) depended on the angle between the center and surround orientations. The strongest suppression was induced when center and surround orientations were parallel. Moreover, the surround influence of the gLFP exhibited an asymmetric spatial organization. These results demonstrate that the gLFP has similar but not identical surround modulation properties, as compared to the spiking activity. The spatiotemporal integration of LFP implies that the oscillation and synchronization of local synaptic inputs may have important functions in surround modulation.

## Introduction

The receptive field surround, which cannot elicit spikes when stimulated alone, can modulate the visual response of the classical receptive field (CRF) when they are stimulated together [1], [2]. The surround modulation of spiking activities has been shown to be mostly suppressive [3]–[6], selective to visual features [7]–[10], and occasionally asymmetrically distributed [11]–[13]. This phenomenon is believed to be the basis of higher functions such as feature integration and figure-ground segregation [14], [15].

Previous studies on surround modulation have mostly focused on spiking activity, whereas another important neural signal – local field potential (LFP) is seldom explored in the center-surround interaction. LFP is the slow component (<250 Hz) of extracellular potentials. This potential is the result of the spatial and temporal summation of different electrical events on membranes of a local cell population. It was estimated that synaptic activities, intrinsic membrane oscillations, and spike after potentials, were the main contributors in the LFP of regular-structured brain tissues such as the neocortex [16], [17]. Thus, the LFP signal may provide different information from that of spiking with respect to the underlying computation being implemented in local circuits. The tuning of direction [18], orientation [19], and contrast [20] were prominent in the LFP gamma band and mostly similar to that of spiking. However, a few investigations showed that the LFP gamma mean power (gLFP) was enhanced, contrary to suppression of spiking, when the stimulus encroached more receptive field surround [21]–[23]. This distinct nature of the tuning properties of LFP may provide new clues regarding the mechanisms underlying surround modulation. Nevertheless, the lack of comprehensive evidence on the surround modulation characteristics of LFP has prevented us from taking full advantage of the LFP signal.

Here, we examined the properties of LFP surround modulation and compared it to those of spiking. Our results showed that LFP modulation mainly occurred along the gamma band and the gamma mean power was suppressed by surround stimulation. Moreover, we found that the surround suppression of the gLFP was orientation-dependent, and the orientation selectivity was similar to that of spiking. Finally, we showed that the gLFP exhibited asymmetric surround suppression distributions. We interpreted the origin of our LFP signal and discussed its possible implications.

## Materials and Methods

### Ethics Statement

Appropriate measures were observed to minimize pain and discomfort, in compliance with the US National Institutes of Health guidelines on the care and use of laboratory animals. All experimental procedures were approved by the Institutional Animal Care and Usage Committee (IACUC) of the Institute of Biophysics, Chinese Academy of Sciences (ID: SYXK(PTJ)2008-114).

### Animal Preparation and Maintenance

A detailed description of the procedures used in this study has been previously reported [24]. Briefly, 15 normal adult cats (2–3 kg) were prepared for extracellular recording. Anesthesia was induced with ketamine hydrochloride (20–30 mg/kg). The continuous infusion of gallamine triethiodide (10 mg/kg/h), sufentanil (0.2 µg/kg/h), and propofol (2–6 mg/kg/h) in Ringer’s solution, were performed in the presence of artificial ventilation to maintain a stable anaesthetized state. The body temperature (38°C), end-tidal CO_2_ (3.5–4%), electrocardiogram (180–240 beats/min), and sometimes electroencephalogram, were continuously monitored. The pupils were dilated with homatropine, and the nictitating membranes were retracted with phenylephrine hydrochloride. The eyes were protected using contact lenses of the appropriate refractive power and covered with 4 mm artificial pupils. The location of the area centralis and optic disks were occasionally checked using a reversible ophthalmoscope. Craniotomy was performed at the Horsley-Clarke coordinates P0–P6 and L0.5–L4 to target the area of the primary visual cortex that represents the central visual field.

### Data Acquisition

Tungsten microelectrodes (FHC Inc., 2–3 MΩ at 1 kHz) were inserted perpendicular to the cortical surface. The reference electrodes were copper wires placed under the dura, at 3–5 mm away from the recording electrodes. The electrodes were advanced using a hydraulic microdrive (Narishige, Japan). The extracellular potentials were pre-amplified and digitized (12 kHz) before they were sent to TDT System-3 RA16 processors (Tucker-Davis Technologies, Inc., USA) for filtering and recording. The spikes were extracted by band-pass filtering (Second-order Butterworth) raw signals between 300 Hz and 3000 Hz; after which, the waveform segments that exceeded 5 times the root mean square were cut. The local field potentials were extracted by band-pass filtering (Second-order Butterworth) the raw signals between 1 Hz and 200 Hz, and sampling at 3 kHz.

### Visual Stimulation

Stimuli were generated by StiLib (version 1.0.8, http://stilib.codeplex.com) and were displayed on a cathode-ray-tube monitor (HM204DTA, Iiyama) at 1024 × 768 resolution in full screen and 120 Hz refresh rate with vertical synchronization. The display was gamma corrected and placed 57 cm from the cat eyes. The ipsilateral eye was covered, and all stimuli were presented to the contralateral eye.

The receptive field location, receptive field size, preferred orientation, spatial frequency, spatial phase, direction, and temporal frequency of the visual responsive cell were initially tested manually, then quantitatively. The accurate position and spatiotemporal organization of the receptive field were measured using standard reverse-correlation procedures [25]. The orientation, spatial frequency and spatial phase tuning were measured by subspace reverse-correlation [26], [27]. Sinusoidal gratings had a 100% Michelson contrast and an average luminance of 20–25 cd/m^2^, which was the same as the uniform background.

Size tuning was tested by linearly enlarging the optimal grating from 0° to 10°, with 0.5° steps. Additional grating sizes of 11°, 13°, 17°, and 25° were added during some of the tests. Stimuli were presented pseudo-randomly every 600–1000 ms, interleaved by 400–500 ms of the uniform background. Each stimulus was repeated for 10–15 times.

**Figure 1:**
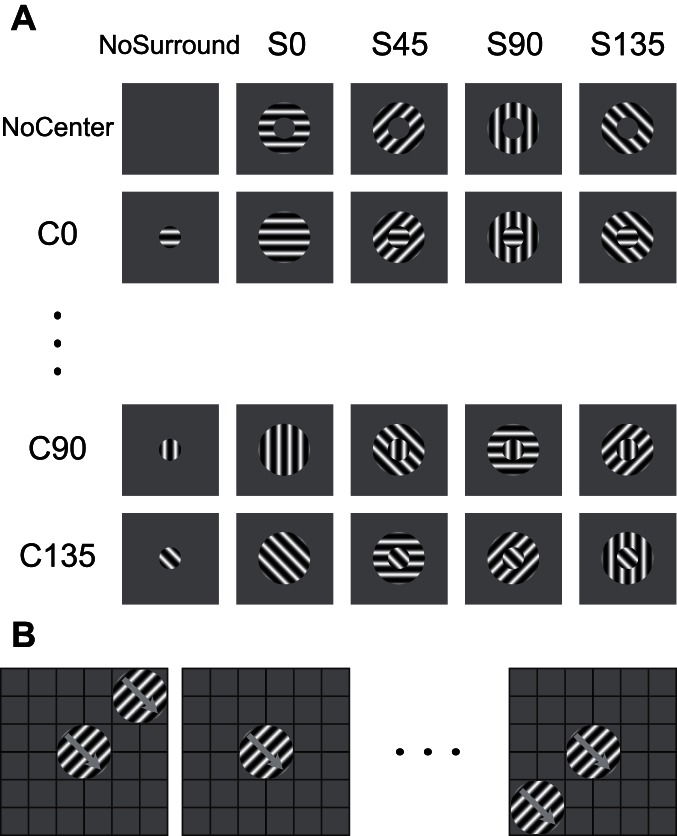
Stimulus paradigm. (A) Center-surround compound stimulus set. The center orientation selectivity was tested under different surround orientations in each column. The orientation selectivity of surround suppression was tested under different center orientations in each row. Surround orientation was the relative angle to the center orientation. (B) Spatial organization of surround modulation was tested by co-stimulation of grid center and other positions. The center stimulus response was chosen as the control level of surround suppression. See text for details.

Once a surround suppressive cell was confirmed through size-tuning curve, the stimulus size that elicited the maximum firing rate (spatial summation field, SSF) was chosen as the center stimulus size, and the smallest size that substantially suppressed the center response was chosen as the surround stimulus size. Optimal center and surround gratings with different orientations were combined (Figure 1A) to test the orientation selectivity of surround suppression. The surround orientation was set as the relative angle difference to the center orientation. The static compound stimuli were presented every 600 ms, interleaved by 400 ms of the uniform background and repeated for 12–15 times.

The spatial structure of surround suppression was tested by the co-stimulation of the center and different surround positions at a square grid (Figure 1B) centered on CRF. The grid size was chosen the same as that of the grating that produced maximum suppression. The center and surround stimuli diameters, as well as the grid interval, were all the same as that of the SSF. The optimal drifting gratings were displayed for 500–600 ms, interleaved with 300–400 ms of the uniform background and repeated 5–6 times.

### Data Analysis

All data analyses were performed in the Matlab computing environment (MathWorks, Inc. USA). Spectrum analysis was performed using Chronux (version 2.0, https://www.chronux.org) [28]. Circular statistics were performed using the CircStat toolbox (version 2010_Dec_01) [29]. The t-test was used to check whether the response of a stimulus was significant, as compared to that of the control stimulus. Unless explicitly stated, all analyses were performed using the sustained responses, thereby excluding 0–200 ms transient responses.

### LFP Analysis

The different types of noise in the raw LFP should be minimized before the actual analysis. The mean ±4SD was used to test if LFP was affected by the noise caused by muscles or electrical bursts, and then the noise segment was cut. Linear interpolation was subsequently performed to fill in any missing data. The entire trial data set was dropped when more than three noise segments were identified. The 50 Hz line-noise was then removed by fitting the LFP to a sine function and subtracting the fitted noise from the signal (Chronux rmlinesc function).

All spectra were estimated using the multi-taper method [30], [31]. The two parameters of this method are (1) the product (TW) of the taper length (T) and the frequency bandwidth (W), and (2) the number of tapers. The multi-taper method allows for balancing of the resolution and variance. We managed to minimize the variance within the reasonable resolution. Our data segment was typically 400–800 ms long and required a resolution of approximately 4 Hz. Therefore, we set a TW of 2.5 with four tapers.

Spikes detected by the same electrode may contaminate the LFP even after the band-pass filtering. To determine the degree of this contamination, the LFP segments were cut around each spike to exclude the LFP components that originated from spike waveform, and the linear interpolations were used to replace the original ones [32]. The resulting LFP spectra were not significantly different from the original LFP spectrum (Figure S1), thereby demonstrating that the spike contamination was trivial. Therefore, we used the original LFP in all of the subsequent analyses.

The visual response of LFP was defined as the change in the power spectrum relative to the control [20], [33] by:
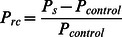
(1)where P_s_ is the LFP power spectrum of the stimulus, and P_control_ is the power spectrum of the uniform background. We also used the z-score [34] definition of the response, but this method did not significantly change the results. Thus, only the results using P_rc_ are shown here.

### Spike Train Power Spectrum

The power spectrum of the spikes was computed using the multi-taper method [35], [36]. Given that discrete sampling would decrease the single-noise ratio, we used a 6 Hz resolution bandwidth and set a TW of 3.5 with six tapers. The raw spectrum was then divided by the mean firing rate [37]. Likewise, we used the auto-correlogram approach to calculate the power spectrum [23], and obtained similar results. Thus, we have only presented the results obtained using the multi-taper method.

### Suppression Index

The degree of surround suppression was represented by the suppression index (SI):
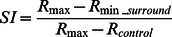
(2)where R_max_ is the maximum response and S_max_ is the corresponding stimulus size. R_min_surround_ is the minimum response elicited by stimuli larger than S_max_, and S_min_ is the corresponding stimulus size. When SI = 0, there is no suppression, and the response is either increasing or reaching a plateau. When SI = 1, the response is suppressed to the level of the control. When SI>1, the response is suppressed to a level lower than that of the control.

### Size-tuning Curve

The size tuning curve was fitted using the difference of gaussian (DoG) function [38], [39] as follows:
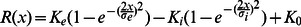
(3)where K_e_ is the amplitude of the excitatory component, and σ_e_ is the width of the excitatory component. K_i_ is the amplitude of the inhibitory component, and σ_i_ is the width of the inhibitory component. K_0_ is the constant offset. The goodness of fit was assessed using Adjust-R^2^. We found the DoG model did not always coincide with the LFP responses (Figure S2). Therefore, the characteristic parameters of size tuning were calculated directly from the response. No data exclusion was performed based on the goodness of fit.

### Surround Suppression Distribution

The asymmetrical distribution of the surround suppression was represented by the asymmetry vector (AV) [11], [12]. First, we computed the suppression vector sum (SVS) using the following equation:
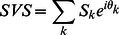
(4)where S_k_ is the suppression amplitude of k_th_ point in the stimulus grid (Figure 1B), and θ_k_ is the angle between the origin to k_th_ point line and the horizontal right line. The angle of SVS is designated as the asymmetry direction (AD). Second, we calculated the asymmetry suppression index (ASI) by normalizing the SVS, as follows:
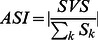
(5)When ASI = 0, the surround suppression is uniformly distributed. When ASI = 1, the suppression comes only from the direction of AD. Finally, AV was constructed, with ASI as its amplitude and AD as its angle. We also computed the axial asymmetry vector (AVa) using equation (4) and (5), but doubling the value of θk. ASIa = 1 indicates that the suppression is distributed on an axis with the direction ADa. When ASIa = 0, the surround suppression is equally distributed on each axis.

## Results

We successfully acquired 75 recording sites. The majority of the recording sites (Figure S3) were presumably from the superficial layer of the cortex.

### Size Tuning

**Figure 2:**
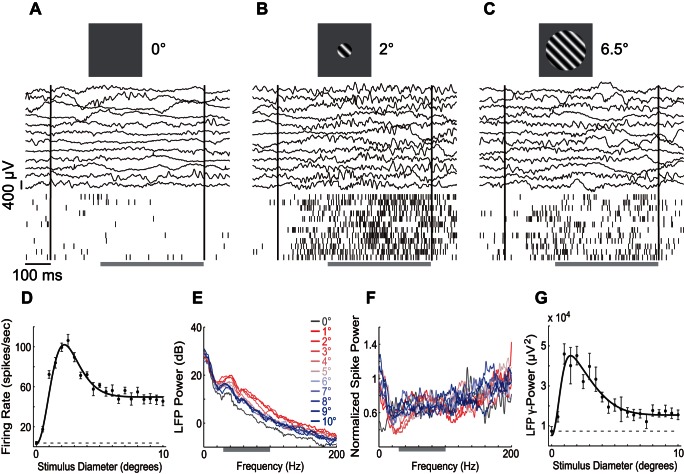
Size-tuning responses of a sample recording site. (A–C) Multi-unit and LFP responses to the enlarging drifting gratings. Black vertical lines indicate the stimulus onset and offset times. The gray horizontal bars indicate the sustained response windows. (D) Size-tuning curve of the sustained response of mean firing rate. The data points indicate the mean ± SE. The solid line is the DoG function fitted from the data, with Adjust-R^2^ = 0.951. The dashed line indicates the response level of uniform background control. (E) LFP power spectrum of different stimulus sizes. The gray bar indicates gamma band. (F) Normalized spike power spectrum of different stimulus sizes. The color code is identical to that of (E). (G) Size-tuning curve of sustained gLFP. The Adjust-R^2^ of the fitted DoG was 0.871. The legends are identical to those of (D).

The size-tuning curve is an effective indicator of the nature and spatial extent of surround modulation. Figure 2 shows the multi-unit and LFP responses in a recording session of the size-tuning test. When the stimulus size was almost equal to the size of the CRF, spiking activity was strong and clear oscillatory activity was visible in the LFP (Figure 2B). When the stimulus size increased beyond the CRF, the spiking activity and LFP oscillation were reduced (Figure 2C). We used the sustained responses of spiking and LFP to characterize the tuning profile. The mean firing rate (MFR) tuning curve (Figure 2D) showed that the optimal stimulus size S_max_ = 2.5°, the most suppressive stimulus size S_min_ = 10°, and the suppression index SI = 0.59.

We transformed LFP to the frequency domain to determine the band where it was modulated by the surround stimulus. Figure 2E shows the power spectra with increasing stimulus sizes. Surround modulation mainly occurred in frequencies higher than 15 Hz, and the surround stimulation generally induced less power than CRF stimulation. Using the mean power of gamma band (30–100 Hz), we acquired another tuning curve (Figure 2G) which had S_max_ = 1°, S_min_ = 7.5°, and SI = 0.88, which was larger than that of the MFR. By contrast, the spike oscillation (Figure 2F) showed no systematic change in the surround stimulation.

**Figure 3:**
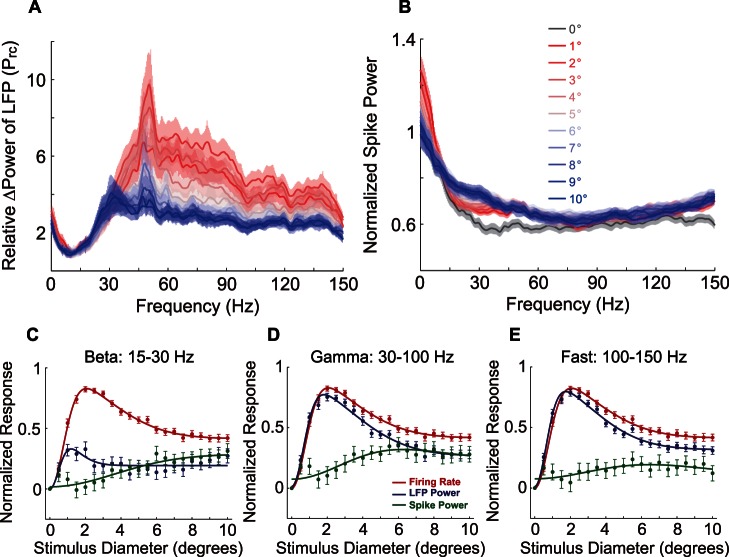
Population average responses of increasing stimulus size. (A) Relative change of LFP power spectra with increasing stimulus size. For the sake of illustration, the data were smoothed by a 5 Hz Sgolay filter. The color shade stripes indicate the standard error. (B) Normalized spike power spectra of different stimulus sizes. The legends are identical to those of (A). (C–E) Size-tuning curves of mean power of LFP (blue) and spikes (green) at different bands, along with that of mean firing rate (red). The data points are the population-averaged responses and their standard error. Solid lines indicate the fitted DoG function. The Adjust-R^2^s of LFP power were 0.523, 0.973, and 0.978 in beta, gamma, and fast band, whereas those of spike power were 0.801, 0.747, and 0.353 in the three bands, respectively. The Adjust-R^2^ of firing rate was 0.992.

The population-averaged surround modulatory effect on LFP and spike train power are shown in Figure 3. The relative power change P_rc_ of LFP was modulated in frequencies higher than 20 Hz, and the normalized power of spiking was modulated from 15 Hz to 40 Hz. Accordingly, we divided the frequency spectrum into three bands, and averaged the size tuning curves of firing rate, mean power of LFP, and mean power of spike train in corresponding band. In the beta band (15–30 Hz, Figure 3C), the LFP mean power (LMP) had a moderate surround suppression with S_max_ = 2°, S_min_ = 4.5°, SI = 0.62. However, the spike mean power (SMP) monotonically increased with the surround size, with SI = 0°, S_max_ = S_min_ = 10°. In the gamma band (30–100 Hz, Figure 3D), the LMP showed clear surround suppression as well as MFR. Moreover, SI of the LMP (SI = 0.65) was larger than that of MFR (SI = 0.49). All other tuning properties were similar, except for their SI. S_max_ of both responses was 2°. S_min_ of LMP and MFR were 7.5° and 9°, respectively. Nevertheless, the SMP response was different to LMP and MFR. SMP increased towards the maximum, at S_max_ = 6°, and slightly decreased towards the minimum surround response at S_min_ = 8.5°, with SI = 0.28. The gamma band was further separated into low (30–60 Hz) and high (60–100 Hz) sub bands. The results did not exhibit obvious differences from that of the whole gamma band (Figure S4). In the fast band (100 150 Hz, Figure 3E), the LMP was largely similar to that in the gamma band, SI = 0.61, S_max_ = 2°, S_min_ = 10°. Nevertheless, the SMP response showed no significant size tuning.

**Figure 4:**
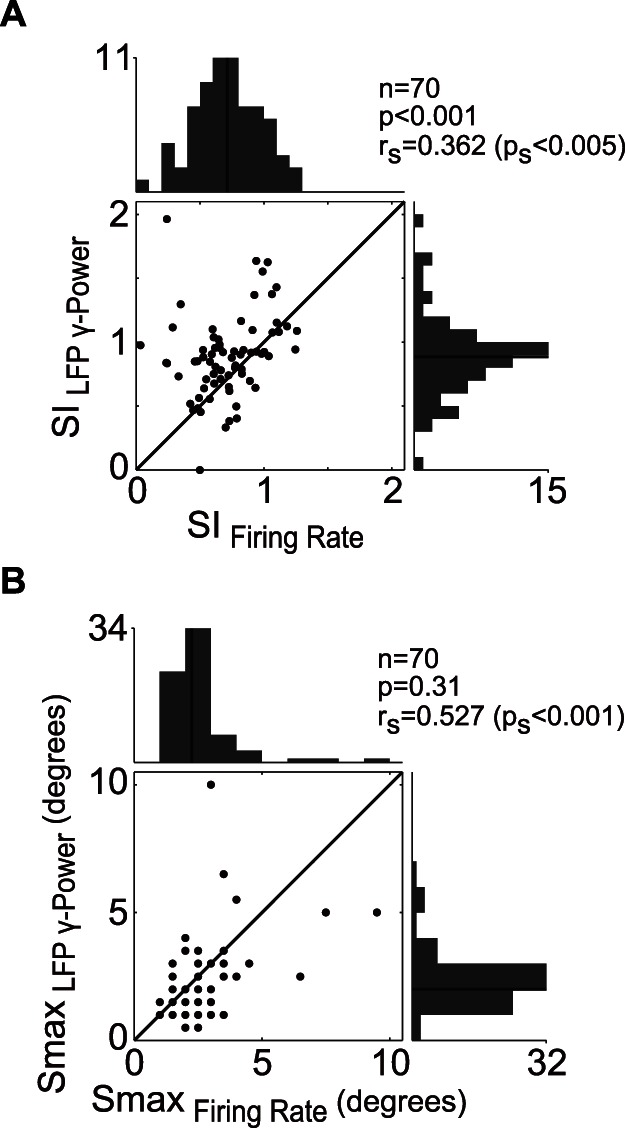
Comparison of firing rate and gLFP surround suppression characteristics. (A) Pairwise comparison of suppression indexes. Black lines in histograms represent the median. The two distributions were significantly different and correlated (Wilcoxon signed-rank test, r_s_ is the Spearman rank correlation coefficient). (B) Distributions of stimulus size that produce maximum response. No significant differences were observed, although, they were significantly correlated. The legends are identical to those in (A).

The LFP gamma band was most sensitive to the stimulus. Thus, we performed a pairwise comparison of the size tuning properties of MFR and gLFP (Figure 4). The SI distribution of the two responses were significantly different (P<0.001, Wilcoxon signed-rank test) and weakly correlated (r_s_ = 0.362, Spearman rank correlation coefficient, P_s_<0.005). The mean SI and corresponding SD of gLFP and MFR were 0.89±0.33 and 0.71±0.26, respectively. However, the two S_max_ distributions were not significantly different (P = 0.31, Wilcoxon signed-rank test), although they were significantly correlated (r_s_ = 0.527, P_s_<0.001). The mean S_max_ and corresponding SD of gLFP and MFR were 2.4° ±1.5° and 2.5° ±1.4°, respectively.

### Orientation Selectivity of Surround Suppression

**Figure 5:**
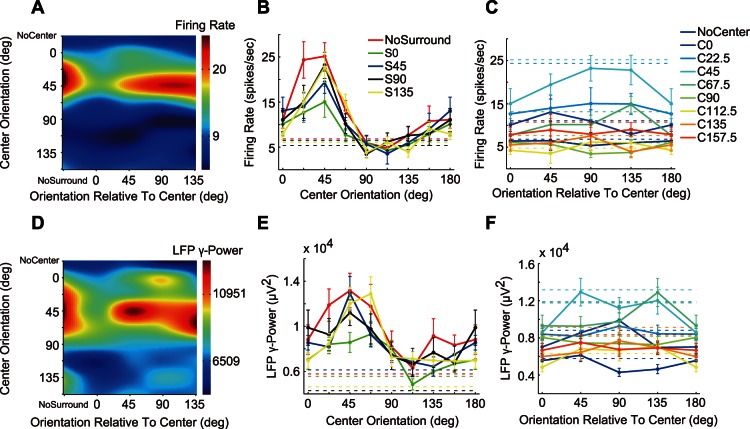
Responses of a sample recording site to the center-surround compound stimulus set. The upper row (A–C) shows firing rate responses and the lower row (D–F) shows the gLFP responses. (A, D) Response of center-surround compound stimulus set. For the sake of illustration, data were interpolated four times by a 2D third-order spline. (B, E) Center orientation tuning curves of firing rate and gLFP under different surround orientations. The data points represent the mean ± SE. The dashed lines indicate responses with no center stimulus. (C, F) Orientation tuning of surround suppression under different center orientations. The dashed lines indicate responses with no surround stimulus.

The surround suppression of the firing rate is dependent on orientation difference between the surround stimulus and the CRF stimulus [7], [8]. However, the surround orientation selectivity of LFP has not been previously reported. We used the center-surround compound stimulus (Figure 1A) to examine whether the LFP surround suppression is orientation-dependent. The multi-unit firing rate and gLFP of a recording site in response to the compound stimuli is presented in Figure 5. First, we analyzed the center orientation tuning under different surround orientations. The optimal center orientation obtained using MFR (Figure 5B) was approximately 45°, and the surround stimulation did not fundamentally change the shape of the tuning curves. The gLFP (Figure 5E) demonstrated center orientation tuning similar to those of MFR. However, the tuning width of gLFP appeared larger than that of MFR. The surround orientation tuning under different center orientations was then analyzed. The center orientation responses of both MFR and gLFP were modulated by the surround orientations (Figure 5C, F). Compared to the center stimulus alone, the same surround orientation caused the strongest suppression in both MFR and gLFP, whereas other orientations had smaller suppressive effects.

**Figure 6:**
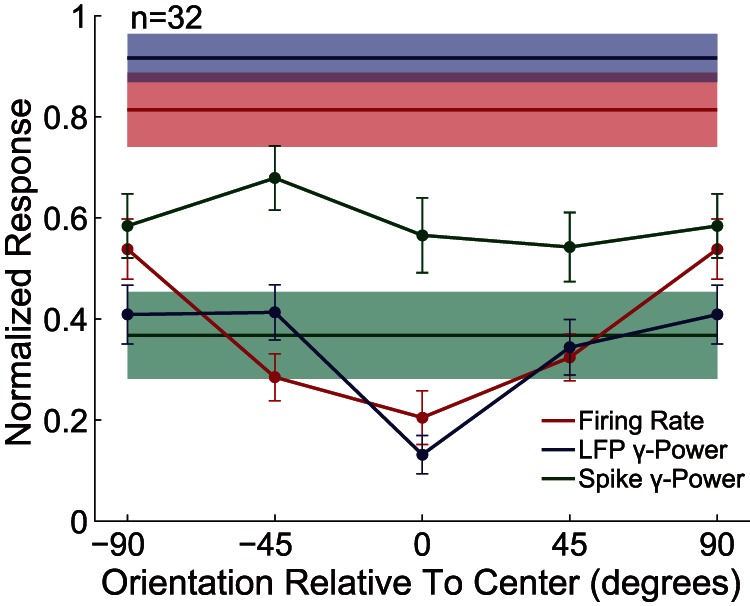
Population-averaged orientation tuning of surround modulation. Horizontal lines and shadings indicate the mean ± SE of CRF stimulation without the surround stimulus. The Data points represent the mean ± SE under different surround orientations.

To obtain the population responses, we normalized the surround orientation tuning curves under different center orientations. These normalized curves were then averaged across center orientations and across the recording sites. The additional surround stimulus suppressed both MFR and gLFP from the response level of the center stimulation alone (Figure 6). Moreover, the degree of suppression was dependent on the difference between the center and surround orientations. Compared to their orthogonal difference, the same orientation caused a 41% decrease for MFR (P<0.001,Wilcoxon signed-rank test) and a 43% decrease for gLFP (P<0.002,Wilcoxon signed-rank test). By contrast, the spike gamma power demonstrated significant surround facilitation, but was not orientation-dependent.

### Spatial Distribution of Surround Suppression

**Figure 7:**
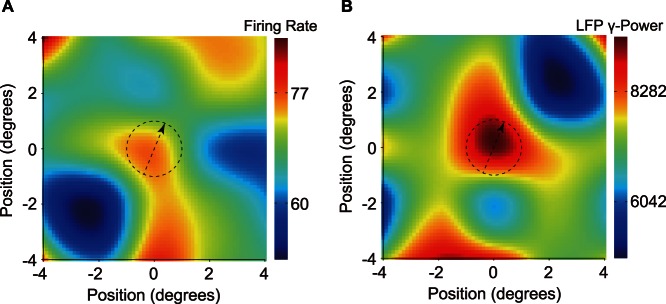
Spatial distribution of surround suppression of a sample recording site. Surround suppression was not always uniform for the firing rate (A) and gLFP (B). In this recording site, both responses had a distinct LSR of similar sizes. These LSRs did not overlap, but were aligned to the optimal direction. The dashed circle indicates the SSF size and position. The dashed arrow indicates the optimal direction of firing rate, which was also the drifting direction of all stimuli. For the sake of illustration, the data were interpolated four times by a 2D third-order spline.

The spatial distribution of surround suppression was investigated by the co-stimulation of SSF and the different surround areas (Figure 1B). The modulation of the MFR and gLFP of a recording site in different surround locations is shown in Figure 7. The drifting direction of all gratings was set to 67.5°, which is the optimal direction of CRF. The diameter of all stimuli was 2°, matching the size of SSF. The majority of the surround positions contributed in the suppression, but a strong local suppressive region (LSR) was evident. The LSR of MFR was located on the opposite side of the optimal direction, whereas the LSR of gLFP was at the side of the optimal direction. These two LSRs had similar sizes that were approximately 1.5 times larger than SSF.

**Figure 8:**
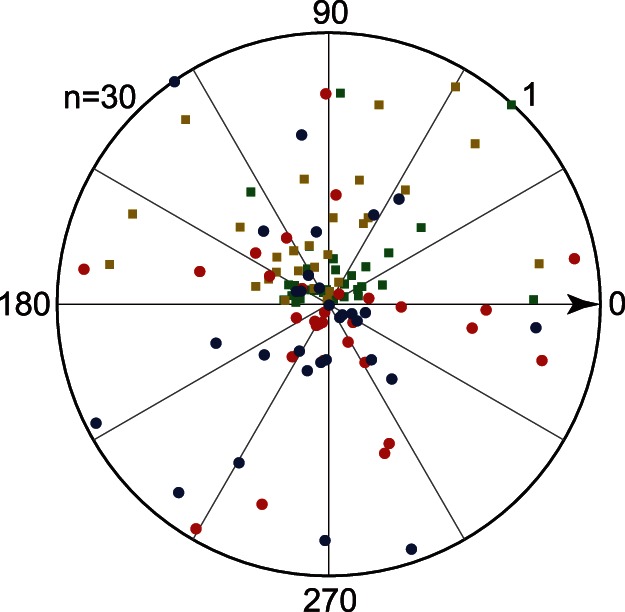
Asymmetry vector and axial asymmetry vector of firing rate and gLFP. Population distributions of the AV of the firing rate (red circle) and gLFP (blue cycle), as well as the AV_a_ of the firing rate (yellow square) and gLFP (green square). The AV angle was rotated so that the optimal direction always horizontally points to the right (black arrow).

**Figure 9:**
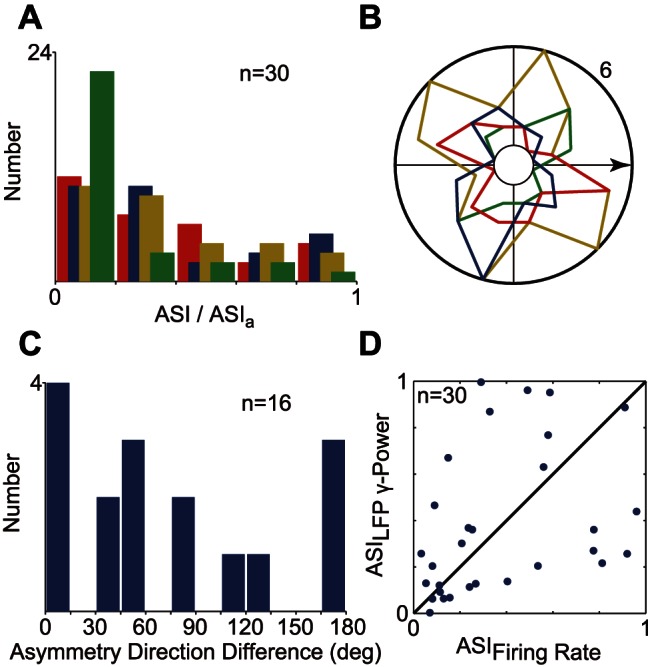
The asymmetric properties of surround suppression. Distributions of ASI, ASI_a_ (A), and AD, AD_a_ (B) are differentiated by color. Red and blue represent asymmetric properties for MFR and gLFP. Yellow and green represent axial asymmetric properties for MFR and gLFP. AD and AD_a_ analyses were only performed when corresponding ASI or ASI_a_ was larger than 0.2. The horizontal arrow indicates the optimal direction. (C) AD difference between the MFR and gLFP when the corresponding ASIs were both larger than 0.2. (D) ASI correlation of MFR and gLFP (r = 0.36, P = 0.048, t-test).

We used asymmetry vector to indicate the properties of surround suppression distribution. Some sites merely showed suppression asymmetry in our recordings, whereas others clearly showed asymmetrical surround suppression (Figure 8). The distribution of ASI and ASI_a_ are presented in Figure 9A. The gLFP had negligible axial asymmetric suppression, because the median of its ASI_a_ was 0.14. The other ASI medians were all approximately 0.26, thereby indicating that almost half of the population had minimal asymmetrical suppression. Thus, we excluded the almost uniformly distributed surround, and the recording sites with ASI>0.2 were chosen for the analysis of the AD. The LSR of gLFP had the tendency to be located at the optimal orientation line, although, this trend was not statistically significant (Figure 9B). By contrast, the AD_a_ of MFR was significantly distributed on the orthogonal cross lines (P = 0.008, circular Rayleigh test) that were aligned close to the optimal orientation and direction lines. Furthermore, we examined the relationship of the asymmetry suppression between MFR and gLFP. The distribution of AD difference between MFR and gLFP are illustrated in Figure 9C. The recording sites wherein ASI>0.2, had 44% of ADs that either overlapped (25%) or were collinear (19%), whereas 19.7% were orthogonal. In addition, the ASI of MFR was significantly correlated to that of gLFP (r = 0.36, P = 0.048, t-test).

## Discussion

In the current study, we recorded local field potential and spiking activity simultaneously in anesthetized cats to investigate the surround modulation characteristics of LFP and to compare it to those of firing rate and spike oscillation. Our results can be summarized as follows: (1) The LFP power was suppressed by surrounding stimulus at the gamma band (30–100 Hz), and the size tuning properties of gLFP were similar to those of the firing rate. (2) The spike oscillation power was enhanced by surround stimulation at the beta band (20–30 Hz) and reached a plateau at the gamma band. (3) The surround suppression of gLFP was orientation-dependent, which is similar to that of the firing rate. Meanwhile, the surround facilitation of spike oscillation was not dependent on orientation. (4) The observed gLFP had both uniform and asymmetric surround suppression distributions.

### Size Tuning of Local Field Potential

There have been a few studies, which investigated the LFP surround modulation [21]–[23]. Gieselmann et al., based on the study using awake monkeys, concluded that the gLFP was surround facilitative [23]. However, their results (Figure 4D) showed that approximately 19% (23/123) of the recordings were surround suppressive (SI ≥0.2; The control response is not subtracted in their definition of SI. Thus, their SI is less than ours provided the same data set). Meanwhile, 11% (8/70) of our recordings exhibited minor or no surround suppression (SI ≤0.5, Figure 4A). The dominant of facilitation in their samples and suppression in ours may be attributed to the different animal states. Propofol, the anesthetic we used, can act on GABA_A_ receptors to prolong the decay time-constant of IPSC [40], [41]. Consequently, the tonic inhibitory current is increased and the amplitude and frequency of oscillatory inhibitory current are decreased during spatiotemporal integration of currents in intracellular and extracellular space. The inhibitory network was believed to be the main substrate that mediated gamma oscillation [42], [43]. Thus, the relative contribution of the oscillation mediated by the inhibitory network in our LFP gamma band may be substantially smaller than that recorded in the awake preparations.

By excluding possible effects on gamma oscillation caused by visual attention [44], [45], Ray and Maunsell [22] found that the relatively low gamma power (30–80 Hz) increased with increasing stimulus size in awake monkeys. However, the relatively high gamma power (>80 Hz) was closely correlated to spiking activities and exhibited surround suppression. According to Ray and Maunsell, spike-associated LFP transients could dominate the LFP at frequencies as low as 50 Hz. However, the low gamma power (30–60 Hz) in our results was not negatively correlated to the high gamma power (60–100 Hz, Figure S4) or fast band power (100–150 Hz, Figure 3). Therefore, the underlying mechanisms of surround facilitating low gamma power observed in awake monkeys were probably suppressed in our anesthetized preparation.

Bauer et al. [21] used anesthetized cats and reported the occurrence of surround facilitation in area 17 and 18. However, their study used binocular visual stimulation, whereas only contralateral eye was stimulated in our study. The facilitative effect they observed may be the consequence of binocular stimulation, which generally elicits stronger oscillations than monocular stimulation [46]–[48]. Halothane, the anesthetic they used, has been confirmed to have more complex effects [49], [50] than propofol. Thus, the use of this drug could be another potential cause of the facilitative effect in their study.

Previous studies showed that hemodynamic signals have a strong correlation with high-frequency LFP oscillation [34] [51]. Examples of these signals are the functional magnetic resonance imaging (fMRI) blood-oxygen-level-dependent (BOLD) signal and the intrinsic optic imaging signal. These correlation studies were similarly conducted in anesthetized preparations. Thus, these hemodynamic signals, based on our result, will be suppressed by surround stimulus. Lippert et al. [52] and Bartolo et al. [53] recently showed that certain stimulating paradigms could induce a negative correlation between the firing rate and gLFP. Moreover, the simultaneously acquired fMRI BOLD signal followed gLFP. These two studies were conducted in awake monkeys. Therefore, the fMRI BOLD signal was related to the gLFP in both the awake and anesthetized states, and in presence of both positive and negative correlations between spiking activity and gLFP. However, the investigations of surround modulation using fMRI [54], [55], intrinsic imaging [56], or magneto-encephalography (MEG) [57], all showed the surround suppression effect, which is consistent with our results. The lack of contradictory fMRI results implies that a more complicated relationship exists between spiking, gLFP, and BOLD. Further studies are necessary to determine the critical factors affecting the relationships among these signals.

### Size Tuning of Spike Oscillation

Consistent with the previous studies [21], [23], Spike oscillation was enhanced when the firing rate was suppressed. In previous studies, the spike oscillation power increased at the same narrow gamma band where the LFP power increased. However, the spike oscillation in this study mainly increased at a lower beta band. The increased spike oscillation was probably caused by the increasing membrane potential oscillation, which narrowed the probable firing window at the peak of membrane oscillation [58]. None of the few intracellular recordings of surround modulation reported direct membrane oscillation [59], [60]. However, Ozeki et al. [61] implied that the membrane potential may oscillate under surround stimulation (Figure 1). Given that the inhibitory network was believed to mediate gamma oscillations, the spike oscillation and the possible membrane potential oscillation may primarily originate from inhibitory network oscillations. Furthermore, changes in the inhibitory network induced by propofol may explain the observed lower frequency and moderate increase of spike oscillation in our results, as compared to previous studies. The regular, structured spiking may be important for neural computation because it can serve as the general synchronization framework for coordinating the different parts of the visual scenes.

### Origin of Oscillations and its Possible Implications

Many studies have suggested that LFP mainly reflect synaptic activities and membrane potential oscillations. However, the contribution of different sources could substantially vary because of the network structure, temporal pattern, or functional regime [17]. Gamma band oscillation was believed to originate primarily from inhibitory network, and the anesthetization in our study may have affected the inhibitory network, reducing its oscillation amplitude and frequency. Thus, the excitatory synaptic activities may become the prominent component in gamma band LFP. Furthermore, the surround suppression of our gLFP was orientation-dependent. This observation indicates that the gLFP might mainly reflect excitatory synaptic activities that are generally more selective to orientations than inhibitory ones.

Receptive field surround suppression has been understood to be primarily mediated by intra-cortical or feedback connections [62], [63]. The average S_min_ of gLFP and the firing rate were both approximately 8° in our results. Likewise, spike oscillation reached its maximum at approximately 6°–8°. These spatial ranges were roughly three times the mean diameter (2.4°) of SSF and covered all the adjacent non-overlapping RFs of similar size. This trend implies that the surround modulation in our results may mainly come from neighboring hypercolumns.

When the center column and its neighboring columns were both stimulated, the gLFP was decreased. As we discussed, this decrease may mainly come from excitatory inputs. Thus, the local excitatory synaptic activities and their synchronization were significantly reduced. This reduction may further decrease the total excitatory input conductance, which was observed in intracellular recordings [59], [61]. The existence of the asymmetric suppression distribution of gLFP indicates that the asymmetric suppression of spike output may come from asymmetric suppression of excitatory inputs. The overlapped or collinear LSR of the firing rate and gLFP were consistent with the directional extension properties of intra-cortical axon terminals connecting similar orientation columns [64], [65]. Nevertheless, the AD of gLFP could be different from the firing rate. This phenomenon implies that the spatial integration of orientations may involve complex interactions of the center and neighboring columns in both sub-threshold and super-threshold levels.

## Supporting Information

Figure S1 **Population responses of LFP with segments around every spike replaced by linear interpolation.** To determine whether spike waveforms contaminated LFP, we cut the LFP segments around each spike detected by the same electrode with (pre-spike, post-spike) time window, and then used linear interpolation to replace the original LFP segments. The results of (−1, 4) (A) and (−2, 8) (B) were shown. The legends are the same as those in Figure 3A.
(TIF)

Figure S2 **Goodness of fit of the DoG model.** Pairwise comparison of the Adjust-R^2^ of the fitted DoG curves. The mean and SD of Adjust-R^2^ for the firing rate and gamma power were 0.83±0.16 and 0.71±0.2, respectively. The legends are the same as those in Figure 4.
(TIF)

Figure S3 **Distribution of recording depth.** During each electrode penetration, we tried to record every isolatable unit. Therefore, the entire depth expansion may correspond to the entire depth of cortex. The depth ratio between layers is more or less constant. Thus, we estimated that majority of our recording sites were located in superficial layers. The black line indicates the median.
(TIF)

Figure S4 **Surround suppression of low and high gamma power.** (A, B) Population-averaged size-tuning curves. The legends are identical to those of Figure 3C. (C, D) Pairwise comparison of the suppression index. The legends are identical to those of Figure 4. The mean and SD of SI for low and high gamma bands were 0.87±0.34 and 0.89±0.22, respectively.
(TIF)
