# Correction: Surround Modulation Characteristics of Local Field Potential and Spiking Activity in Primary Visual Cortex of Cat

**DOI:** 10.1371/annotation/93ae1c28-98bc-4a65-b28f-d98059011d38

**Published:** 2013-09-23

**Authors:** Li Zhang, Bing Li

Multiple funding organizations and grants were incorrectly omitted from the Funding Statement. The Funding Statement should read: "This work was supported by grants from National Basic Research Program of China (2005CB724301), Hi-Tech Research and Development Program of China (2007AA02Z313), The National Natural Science Foundation of China (90408020), and Key Innovation Project of Chinese Academy of Sciences (KSCX1-YW-R-32). The funders had no role in study design, data collection and analysis, decision to publish, or preparation of the manuscript."

In addition, the second author was not correctly indicated as being a Corresponding Author for the article. Bing Li's email address is: lib@sun5.ibp.ac.cn.

Finally, the second author (BL) was not correctly indicated as having "Contributed reagents/materials/analysis tools." 

