# Supplementary figures and images for: Surround Modulation Characteristics of Local Field Potential and Spiking Activity in Primary Visual Cortex of Cat
Source: PLoS One. 2013 May 15;8(5):e64492. doi: 10.1371/journal.pone.0064492 (PMC3655189; doi:10.1371/journal.pone.0064492)

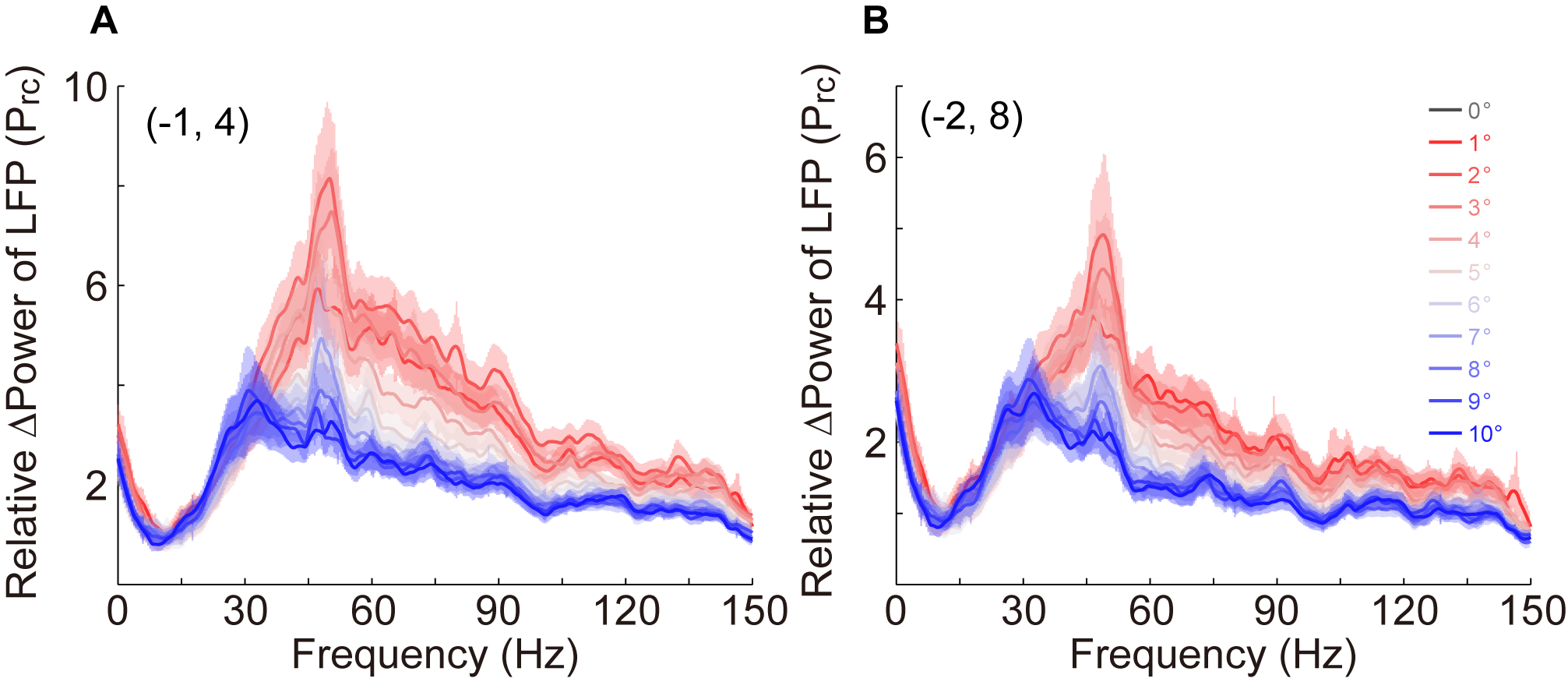

Supplement: Figure S1 — Population responses of LFP with segments around every spike replaced by linear interpolation. To determine whether spike waveforms contaminated LFP, we cut the LFP segments around each spike detected by the same electrode with (pre-spike, post-spike) time window, and then used linear interpolation to replace the original LFP segments. The results of (−1, 4) (A) and (−2, 8) (B) were shown. The legends are the same as those in Figure 3A. (TIF) [file pone.0064492.s001.tif]

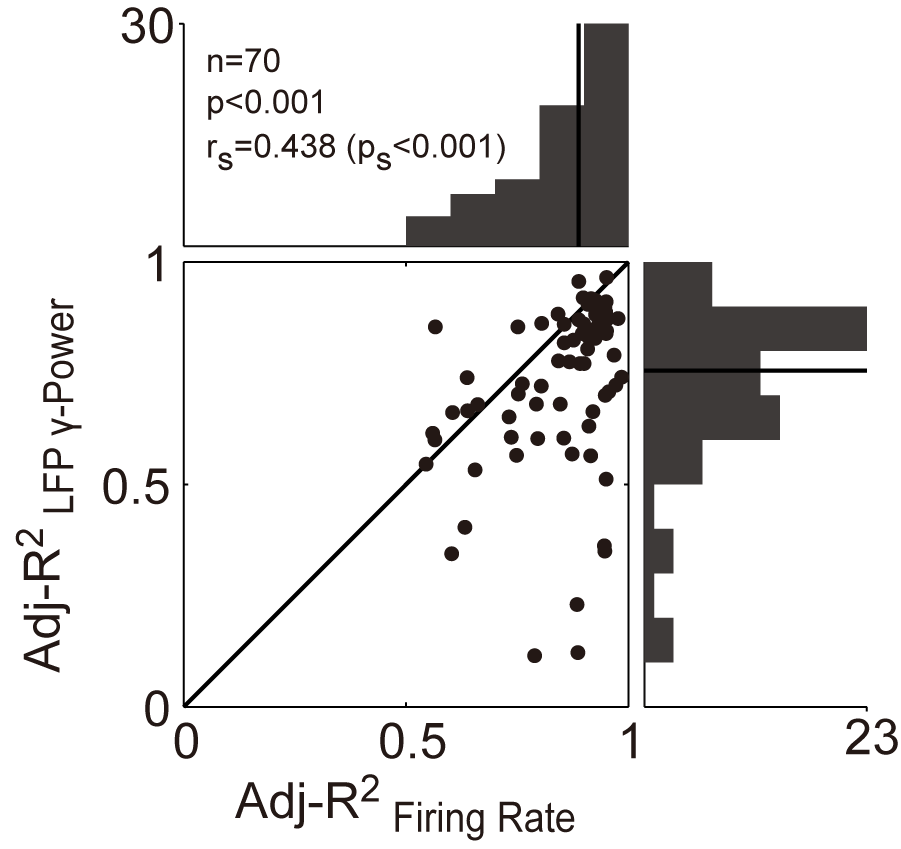

Supplement: Figure S2 — Goodness of fit of the DoG model. Pairwise comparison of the Adjust-R2 of the fitted DoG curves. The mean and SD of Adjust-R2 for the firing rate and gamma power were 0.83±0.16 and 0.71±0.2, respectively. The legends are the same as those in Figure 4. (TIF) [file pone.0064492.s002.tif]

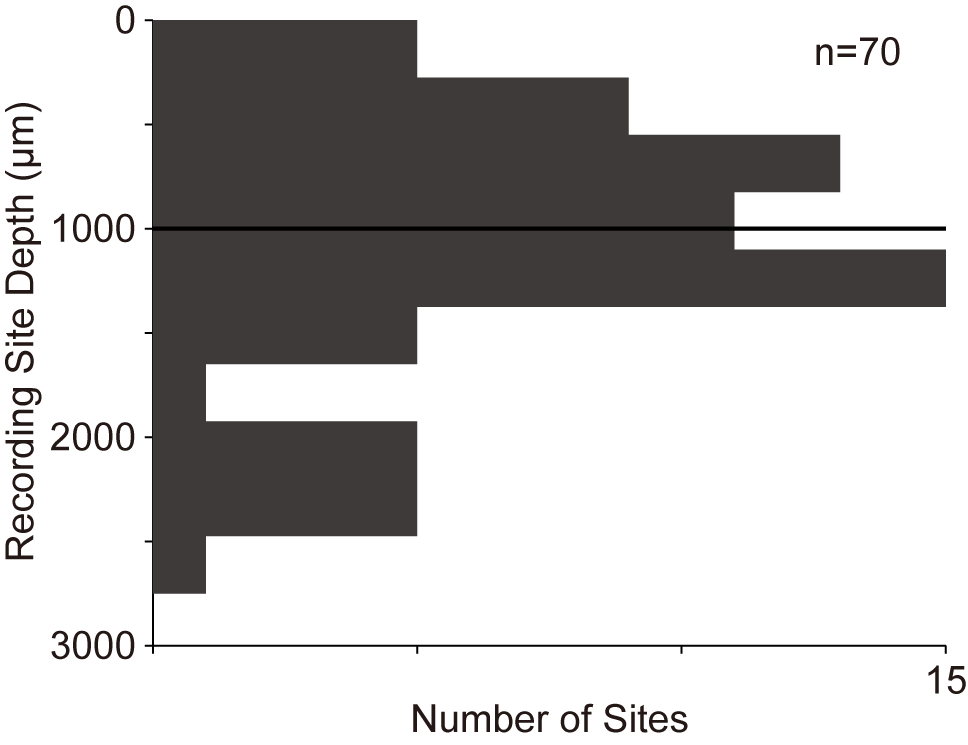

Supplement: Figure S3 — Distribution of recording depth. During each electrode penetration, we tried to record every isolatable unit. Therefore, the entire depth expansion may correspond to the entire depth of cortex. The depth ratio between layers is more or less constant. Thus, we estimated that majority of our recording sites were located in superficial layers. The black line indicates the median. (TIF) [file pone.0064492.s003.tif]

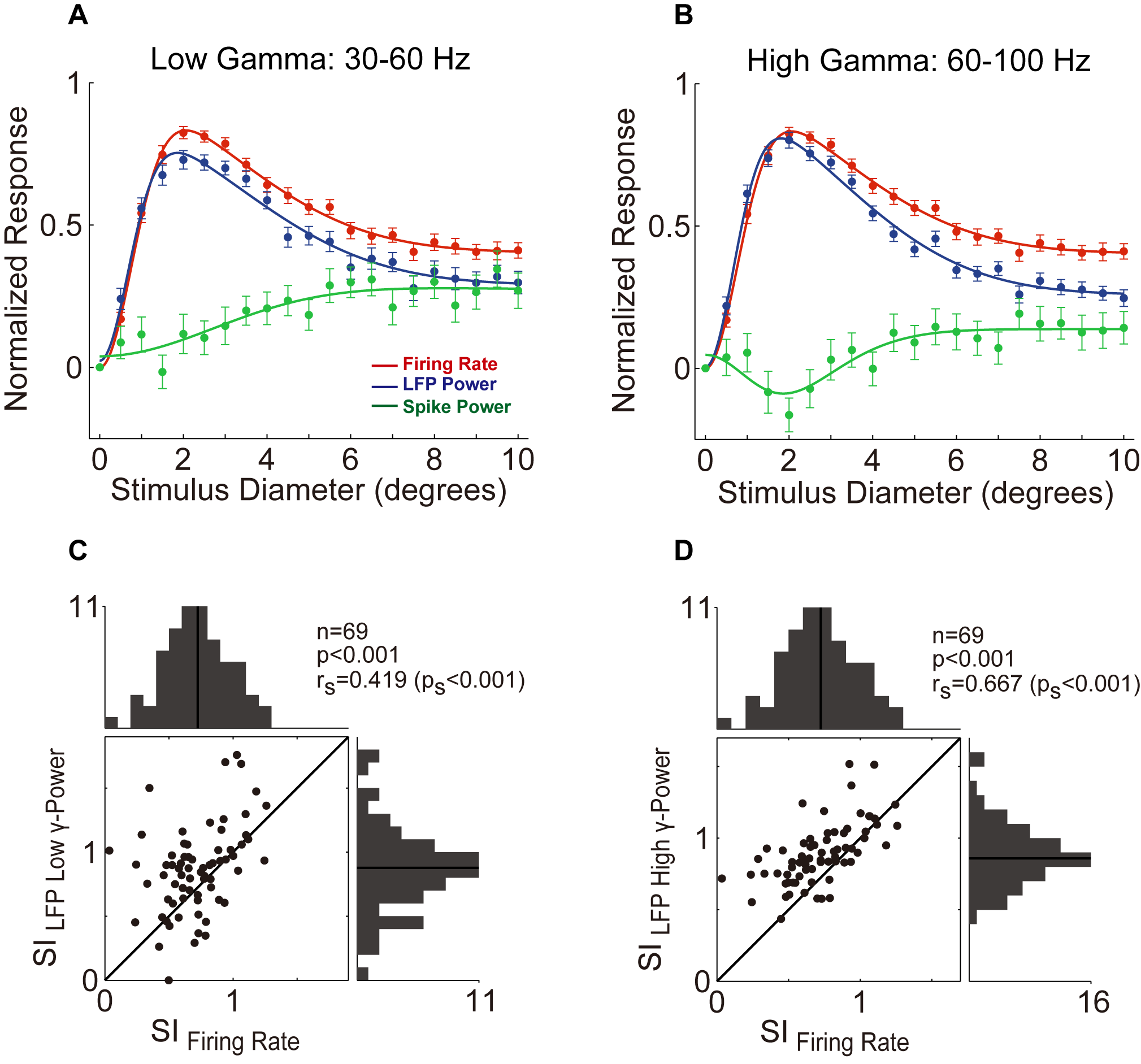

Supplement: Figure S4 — Surround suppression of low and high gamma power. (A, B) Population-averaged size-tuning curves. The legends are identical to those of Figure 3C. (C, D) Pairwise comparison of the suppression index. The legends are identical to those of Figure 4. The mean and SD of SI for low and high gamma bands were 0.87±0.34 and 0.89±0.22, respectively. (TIF) [file pone.0064492.s004.tif]
